# Supplementary material for: Pyruvate Dehydrogenase Kinase 1 inhibition mediated oxidative phosphorylation enhancement in cartilage promotes osteoarthritis progression
Source: BMC Musculoskelet Disord. 2023 Jul 20;24:597. doi: 10.1186/s12891-023-06585-6 (PMC10357736; doi:10.1186/s12891-023-06585-6)
Supplement: Supplementary file 1 — Additional file 1. [file 12891_2023_6585_MOESM1_ESM.zip › BP.Gsea.1653623667859/GOBP_MRNA_PROCESSING.html]

Details for gene set GOBP\_MRNA\_PROCESSING[GSEA]

|  || Dataset | 55457\_collapsed\_to\_symbols.cls.cls #OA\_versus\_normal.cls.cls #OA\_versus\_normal\_repos |
| Phenotype | cls.cls#OA\_versus\_normal\_repos |
| Upregulated in class | normal |
| GeneSet | GOBP\_MRNA\_PROCESSING |
| Enrichment Score (ES) | -0.3423379 |
| Normalized Enrichment Score (NES) | -1.7608368 |
| Nominal p-value | 0.0 |
| FDR q-value | 0.111878395 |
| FWER p-Value | 0.998 |
Table: GSEA Results Summary

  

Fig 1: Enrichment plot: GOBP\_MRNA\_PROCESSING      
 Profile of the Running ES Score & Positions of GeneSet Members on the Rank Ordered List

  

| SYMBOL | TITLE | RANK IN GENE LIST | RANK METRIC SCORE | RUNNING ES | CORE ENRICHMENT || 1 | LSM4 | "LSM4 homolog, U6 small nuclear RNA and mRNA degradation associated [Source:HGNC Symbol;Acc:HGNC:17259]" | 174 | 0.837 | -0.0075 | No |
| 2 | PAN2 | poly(A) specific ribonuclease subunit PAN2 [Source:HGNC Symbol;Acc:HGNC:20074] | 190 | 0.828 | -0.0023 | No |
| 3 | FAM172A | family with sequence similarity 172 member A [Source:HGNC Symbol;Acc:HGNC:25365] | 251 | 0.785 | -0.0011 | No |
| 4 | TXNL4A | thioredoxin like 4A [Source:HGNC Symbol;Acc:HGNC:30551] | 321 | 0.736 | -0.0009 | No |
| 5 | SF3B5 | splicing factor 3b subunit 5 [Source:HGNC Symbol;Acc:HGNC:21083] | 328 | 0.734 | 0.0042 | No |
| 6 | SRPK3 | SRSF protein kinase 3 [Source:HGNC Symbol;Acc:HGNC:11402] | 329 | 0.734 | 0.0099 | No |
| 7 | PPIE | peptidylprolyl isomerase E [Source:HGNC Symbol;Acc:HGNC:9258] | 334 | 0.733 | 0.0152 | No |
| 8 | SNRNP25 | small nuclear ribonucleoprotein U11/U12 subunit 25 [Source:HGNC Symbol;Acc:HGNC:14161] | 354 | 0.723 | 0.0192 | No |
| 9 | RNF113A | ring finger protein 113A [Source:HGNC Symbol;Acc:HGNC:12974] | 355 | 0.723 | 0.0248 | No |
| 10 | LSM3 | "LSM3 homolog, U6 small nuclear RNA and mRNA degradation associated [Source:HGNC Symbol;Acc:HGNC:17874]" | 377 | 0.716 | 0.0286 | No |
| 11 | SNRPN | small nuclear ribonucleoprotein polypeptide N [Source:HGNC Symbol;Acc:HGNC:11164] | 544 | 0.644 | 0.0203 | No |
| 12 | SCNM1 | sodium channel modifier 1 [Source:HGNC Symbol;Acc:HGNC:23136] | 557 | 0.638 | 0.0242 | No |
| 13 | RBM4B | RNA binding motif protein 4B [Source:HGNC Symbol;Acc:HGNC:28842] | 681 | 0.598 | 0.0190 | No |
| 14 | ECD | ecdysoneless cell cycle regulator [Source:HGNC Symbol;Acc:HGNC:17029] | 682 | 0.598 | 0.0236 | No |
| 15 | CLP1 | cleavage factor polyribonucleotide kinase subunit 1 [Source:HGNC Symbol;Acc:HGNC:16999] | 748 | 0.582 | 0.0229 | No |
| 16 | CRNKL1 | crooked neck pre-mRNA splicing factor 1 [Source:HGNC Symbol;Acc:HGNC:15762] | 803 | 0.568 | 0.0229 | No |
| 17 | PRPF18 | pre-mRNA processing factor 18 [Source:HGNC Symbol;Acc:HGNC:17351] | 836 | 0.559 | 0.0247 | No |
| 18 | SRSF9 | serine and arginine rich splicing factor 9 [Source:HGNC Symbol;Acc:HGNC:10791] | 882 | 0.548 | 0.0253 | No |
| 19 | PPP1R8 | protein phosphatase 1 regulatory subunit 8 [Source:HGNC Symbol;Acc:HGNC:9296] | 885 | 0.547 | 0.0293 | No |
| 20 | DDX23 | DEAD-box helicase 23 [Source:HGNC Symbol;Acc:HGNC:17347] | 913 | 0.538 | 0.0313 | No |
| 21 | SUPT4H1 | "SPT4 homolog, DSIF elongation factor subunit [Source:HGNC Symbol;Acc:HGNC:11467]" | 949 | 0.529 | 0.0326 | No |
| 22 | USP39 | ubiquitin specific peptidase 39 [Source:HGNC Symbol;Acc:HGNC:20071] | 991 | 0.519 | 0.0333 | No |
| 23 | SNRPD3 | small nuclear ribonucleoprotein D3 polypeptide [Source:HGNC Symbol;Acc:HGNC:11160] | 1046 | 0.508 | 0.0329 | No |
| 24 | SAP18 | Sin3A associated protein 18 [Source:HGNC Symbol;Acc:HGNC:10530] | 1099 | 0.496 | 0.0325 | No |
| 25 | NELFE | negative elongation factor complex member E [Source:HGNC Symbol;Acc:HGNC:13974] | 1135 | 0.489 | 0.0335 | No |
| 26 | HTATSF1 | HIV-1 Tat specific factor 1 [Source:HGNC Symbol;Acc:HGNC:5276] | 1157 | 0.485 | 0.0356 | No |
| 27 | CD2BP2 | CD2 cytoplasmic tail binding protein 2 [Source:HGNC Symbol;Acc:HGNC:1656] | 1158 | 0.485 | 0.0393 | No |
| 28 | UBL5 | ubiquitin like 5 [Source:HGNC Symbol;Acc:HGNC:13736] | 1160 | 0.485 | 0.0429 | No |
| 29 | MBNL3 | muscleblind like splicing regulator 3 [Source:HGNC Symbol;Acc:HGNC:20564] | 1165 | 0.483 | 0.0463 | No |
| 30 | SNRPD2 | small nuclear ribonucleoprotein D2 polypeptide [Source:HGNC Symbol;Acc:HGNC:11159] | 1290 | 0.460 | 0.0400 | No |
| 31 | GEMIN2 | gem nuclear organelle associated protein 2 [Source:HGNC Symbol;Acc:HGNC:10884] | 1322 | 0.454 | 0.0410 | No |
| 32 | BCAS2 | BCAS2 pre-mRNA processing factor [Source:HGNC Symbol;Acc:HGNC:975] | 1356 | 0.449 | 0.0418 | No |
| 33 | BUD31 | BUD31 homolog [Source:HGNC Symbol;Acc:HGNC:29629] | 1380 | 0.446 | 0.0434 | No |
| 34 | LSM1 | "LSM1 homolog, mRNA degradation associated [Source:HGNC Symbol;Acc:HGNC:20472]" | 1470 | 0.433 | 0.0396 | No |
| 35 | PSIP1 | PC4 and SFRS1 interacting protein 1 [Source:HGNC Symbol;Acc:HGNC:9527] | 1482 | 0.431 | 0.0420 | No |
| 36 | PPIH | peptidylprolyl isomerase H [Source:HGNC Symbol;Acc:HGNC:14651] | 1555 | 0.419 | 0.0395 | No |
| 37 | SNRPA | small nuclear ribonucleoprotein polypeptide A [Source:HGNC Symbol;Acc:HGNC:11151] | 1670 | 0.403 | 0.0335 | No |
| 38 | CLNS1A | chloride nucleotide-sensitive channel 1A [Source:HGNC Symbol;Acc:HGNC:2080] | 1671 | 0.403 | 0.0366 | No |
| 39 | RBM23 | RNA binding motif protein 23 [Source:HGNC Symbol;Acc:HGNC:20155] | 1678 | 0.402 | 0.0392 | No |
| 40 | ARL6IP4 | ADP ribosylation factor like GTPase 6 interacting protein 4 [Source:HGNC Symbol;Acc:HGNC:18076] | 1725 | 0.394 | 0.0385 | No |
| 41 | NUDT21 | nudix hydrolase 21 [Source:HGNC Symbol;Acc:HGNC:13870] | 1734 | 0.393 | 0.0409 | No |
| 42 | BRDT | bromodomain testis associated [Source:HGNC Symbol;Acc:HGNC:1105] | 1812 | 0.382 | 0.0377 | No |
| 43 | GPKOW | G-patch domain and KOW motifs [Source:HGNC Symbol;Acc:HGNC:30677] | 1814 | 0.382 | 0.0406 | No |
| 44 | AICDA | activation induced cytidine deaminase [Source:HGNC Symbol;Acc:HGNC:13203] | 1825 | 0.381 | 0.0427 | No |
| 45 | ADARB2 | adenosine deaminase RNA specific B2 (inactive) [Source:HGNC Symbol;Acc:HGNC:227] | 1828 | 0.381 | 0.0455 | No |
| 46 | YJU2 | YJU2 splicing factor homolog [Source:HGNC Symbol;Acc:HGNC:25518] | 1832 | 0.380 | 0.0482 | No |
| 47 | SYF2 | SYF2 pre-mRNA splicing factor [Source:HGNC Symbol;Acc:HGNC:19824] | 1881 | 0.375 | 0.0472 | No |
| 48 | MFAP1 | microfibril associated protein 1 [Source:HGNC Symbol;Acc:HGNC:7032] | 1911 | 0.371 | 0.0477 | No |
| 49 | RBM4 | RNA binding motif protein 4 [Source:HGNC Symbol;Acc:HGNC:9901] | 1970 | 0.364 | 0.0459 | No |
| 50 | SRSF8 | serine and arginine rich splicing factor 8 [Source:HGNC Symbol;Acc:HGNC:16988] | 2053 | 0.356 | 0.0421 | No |
| 51 | ESRP1 | epithelial splicing regulatory protein 1 [Source:HGNC Symbol;Acc:HGNC:25966] | 2064 | 0.355 | 0.0440 | No |
| 52 | H2AB3 | H2A.B variant histone 3 [Source:HGNC Symbol;Acc:HGNC:14455] | 2090 | 0.351 | 0.0447 | No |
| 53 | DBR1 | debranching RNA lariats 1 [Source:HGNC Symbol;Acc:HGNC:15594] | 2091 | 0.351 | 0.0474 | No |
| 54 | LSM5 | "LSM5 homolog, U6 small nuclear RNA and mRNA degradation associated [Source:HGNC Symbol;Acc:HGNC:17162]" | 2127 | 0.347 | 0.0473 | No |
| 55 | MTREX | Mtr4 exosome RNA helicase [Source:HGNC Symbol;Acc:HGNC:18734] | 2138 | 0.346 | 0.0491 | No |
| 56 | THOC7 | THO complex 7 [Source:HGNC Symbol;Acc:HGNC:29874] | 2170 | 0.341 | 0.0493 | No |
| 57 | FAM50A | family with sequence similarity 50 member A [Source:HGNC Symbol;Acc:HGNC:18786] | 2203 | 0.338 | 0.0493 | No |
| 58 | SCAF8 | SR-related CTD associated factor 8 [Source:HGNC Symbol;Acc:HGNC:20959] | 2207 | 0.337 | 0.0517 | No |
| 59 | ESRP2 | epithelial splicing regulatory protein 2 [Source:HGNC Symbol;Acc:HGNC:26152] | 2435 | 0.310 | 0.0359 | No |
| 60 | AURKAIP1 | aurora kinase A interacting protein 1 [Source:HGNC Symbol;Acc:HGNC:24114] | 2535 | 0.299 | 0.0303 | No |
| 61 | DCPS | "decapping enzyme, scavenger [Source:HGNC Symbol;Acc:HGNC:29812]" | 2570 | 0.295 | 0.0299 | No |
| 62 | RBM7 | RNA binding motif protein 7 [Source:HGNC Symbol;Acc:HGNC:9904] | 2620 | 0.288 | 0.0282 | No |
| 63 | APOBEC1 | apolipoprotein B mRNA editing enzyme catalytic subunit 1 [Source:HGNC Symbol;Acc:HGNC:604] | 2695 | 0.280 | 0.0244 | No |
| 64 | CSTF2T | cleavage stimulation factor subunit 2 tau variant [Source:HGNC Symbol;Acc:HGNC:17086] | 2749 | 0.275 | 0.0223 | No |
| 65 | RAVER2 | "ribonucleoprotein, PTB binding 2 [Source:HGNC Symbol;Acc:HGNC:25577]" | 2956 | 0.254 | 0.0078 | No |
| 66 | FXR1 | FMR1 autosomal homolog 1 [Source:HGNC Symbol;Acc:HGNC:4023] | 3012 | 0.249 | 0.0053 | No |
| 67 | BARD1 | BRCA1 associated RING domain 1 [Source:HGNC Symbol;Acc:HGNC:952] | 3051 | 0.245 | 0.0042 | No |
| 68 | C1QBP | complement C1q binding protein [Source:HGNC Symbol;Acc:HGNC:1243] | 3083 | 0.241 | 0.0036 | No |
| 69 | SF3A1 | splicing factor 3a subunit 1 [Source:HGNC Symbol;Acc:HGNC:10765] | 3152 | 0.235 | -0.0001 | No |
| 70 | GEMIN8 | gem nuclear organelle associated protein 8 [Source:HGNC Symbol;Acc:HGNC:26044] | 3156 | 0.235 | 0.0015 | No |
| 71 | MTPAP | mitochondrial poly(A) polymerase [Source:HGNC Symbol;Acc:HGNC:25532] | 3175 | 0.233 | 0.0019 | No |
| 72 | PUF60 | poly(U) binding splicing factor 60 [Source:HGNC Symbol;Acc:HGNC:17042] | 3237 | 0.227 | -0.0013 | No |
| 73 | PRMT7 | protein arginine methyltransferase 7 [Source:HGNC Symbol;Acc:HGNC:25557] | 3250 | 0.226 | -0.0005 | No |
| 74 | FRG1 | FSHD region gene 1 [Source:HGNC Symbol;Acc:HGNC:3954] | 3254 | 0.226 | 0.0010 | No |
| 75 | ZMAT5 | zinc finger matrin-type 5 [Source:HGNC Symbol;Acc:HGNC:28046] | 3288 | 0.222 | 0.0001 | No |
| 76 | DDX1 | DEAD-box helicase 1 [Source:HGNC Symbol;Acc:HGNC:2734] | 3305 | 0.221 | 0.0005 | No |
| 77 | SON | SON DNA and RNA binding protein [Source:HGNC Symbol;Acc:HGNC:11183] | 3374 | 0.214 | -0.0033 | No |
| 78 | LARP7 | "La ribonucleoprotein 7, transcriptional regulator [Source:HGNC Symbol;Acc:HGNC:24912]" | 3395 | 0.212 | -0.0033 | No |
| 79 | CSTF3 | cleavage stimulation factor subunit 3 [Source:HGNC Symbol;Acc:HGNC:2485] | 3426 | 0.209 | -0.0041 | No |
| 80 | CIRBP | cold inducible RNA binding protein [Source:HGNC Symbol;Acc:HGNC:1982] | 3441 | 0.207 | -0.0036 | No |
| 81 | RPRD2 | regulation of nuclear pre-mRNA domain containing 2 [Source:HGNC Symbol;Acc:HGNC:29039] | 3472 | 0.205 | -0.0044 | No |
| 82 | SNRPF | small nuclear ribonucleoprotein polypeptide F [Source:HGNC Symbol;Acc:HGNC:11162] | 3509 | 0.201 | -0.0057 | No |
| 83 | RBFOX2 | RNA binding fox-1 homolog 2 [Source:HGNC Symbol;Acc:HGNC:9906] | 3523 | 0.200 | -0.0052 | No |
| 84 | MBNL2 | muscleblind like splicing regulator 2 [Source:HGNC Symbol;Acc:HGNC:16746] | 3524 | 0.200 | -0.0037 | No |
| 85 | RALY | RALY heterogeneous nuclear ribonucleoprotein [Source:HGNC Symbol;Acc:HGNC:15921] | 3546 | 0.198 | -0.0038 | No |
| 86 | TSEN34 | tRNA splicing endonuclease subunit 34 [Source:HGNC Symbol;Acc:HGNC:15506] | 3617 | 0.192 | -0.0080 | No |
| 87 | LSM7 | "LSM7 homolog, U6 small nuclear RNA and mRNA degradation associated [Source:HGNC Symbol;Acc:HGNC:20470]" | 3627 | 0.191 | -0.0072 | No |
| 88 | SNRPC | small nuclear ribonucleoprotein polypeptide C [Source:HGNC Symbol;Acc:HGNC:11157] | 3649 | 0.190 | -0.0074 | No |
| 89 | COIL | coilin [Source:HGNC Symbol;Acc:HGNC:2184] | 3684 | 0.187 | -0.0087 | No |
| 90 | SLBP | stem-loop binding protein [Source:HGNC Symbol;Acc:HGNC:10904] | 3703 | 0.185 | -0.0087 | No |
| 91 | KHDRBS3 | "KH RNA binding domain containing, signal transduction associated 3 [Source:HGNC Symbol;Acc:HGNC:18117]" | 3714 | 0.184 | -0.0081 | No |
| 92 | CSTF1 | cleavage stimulation factor subunit 1 [Source:HGNC Symbol;Acc:HGNC:2483] | 3732 | 0.183 | -0.0081 | No |
| 93 | GEMIN6 | gem nuclear organelle associated protein 6 [Source:HGNC Symbol;Acc:HGNC:20044] | 3741 | 0.182 | -0.0073 | No |
| 94 | KHDRBS1 | "KH RNA binding domain containing, signal transduction associated 1 [Source:HGNC Symbol;Acc:HGNC:18116]" | 3748 | 0.182 | -0.0064 | No |
| 95 | ZRSR2 | "zinc finger CCCH-type, RNA binding motif and serine/arginine rich 2 [Source:HGNC Symbol;Acc:HGNC:23019]" | 3771 | 0.179 | -0.0068 | No |
| 96 | SNU13 | small nuclear ribonucleoprotein 13 [Source:HGNC Symbol;Acc:HGNC:7819] | 3795 | 0.178 | -0.0072 | No |
| 97 | CWF19L1 | CWF19 like cell cycle control factor 1 [Source:HGNC Symbol;Acc:HGNC:25613] | 3981 | 0.157 | -0.0208 | No |
| 98 | LSM6 | "LSM6 homolog, U6 small nuclear RNA and mRNA degradation associated [Source:HGNC Symbol;Acc:HGNC:17017]" | 4019 | 0.154 | -0.0226 | No |
| 99 | SNRNP27 | small nuclear ribonucleoprotein U4/U6.U5 subunit 27 [Source:HGNC Symbol;Acc:HGNC:30240] | 4037 | 0.152 | -0.0227 | No |
| 100 | PTCD2 | pentatricopeptide repeat domain 2 [Source:HGNC Symbol;Acc:HGNC:25734] | 4097 | 0.146 | -0.0263 | No |
| 101 | MAGOH | "mago homolog, exon junction complex subunit [Source:HGNC Symbol;Acc:HGNC:6815]" | 4266 | 0.131 | -0.0387 | No |
| 102 | APOBEC2 | apolipoprotein B mRNA editing enzyme catalytic subunit 2 [Source:HGNC Symbol;Acc:HGNC:605] | 4279 | 0.130 | -0.0387 | No |
| 103 | ESS2 | ess-2 splicing factor homolog [Source:HGNC Symbol;Acc:HGNC:16817] | 4347 | 0.124 | -0.0431 | No |
| 104 | A1CF | APOBEC1 complementation factor [Source:HGNC Symbol;Acc:HGNC:24086] | 4368 | 0.123 | -0.0437 | No |
| 105 | GPATCH1 | G-patch domain containing 1 [Source:HGNC Symbol;Acc:HGNC:24658] | 4406 | 0.120 | -0.0458 | No |
| 106 | SNRNP35 | small nuclear ribonucleoprotein U11/U12 subunit 35 [Source:HGNC Symbol;Acc:HGNC:30852] | 4547 | 0.109 | -0.0561 | No |
| 107 | KHDRBS2 | "KH RNA binding domain containing, signal transduction associated 2 [Source:HGNC Symbol;Acc:HGNC:18114]" | 4550 | 0.109 | -0.0554 | No |
| 108 | PRPF6 | pre-mRNA processing factor 6 [Source:HGNC Symbol;Acc:HGNC:15860] | 4567 | 0.108 | -0.0559 | No |
| 109 | PCBP4 | poly(rC) binding protein 4 [Source:HGNC Symbol;Acc:HGNC:8652] | 4577 | 0.107 | -0.0558 | No |
| 110 | LSM2 | "LSM2 homolog, U6 small nuclear RNA and mRNA degradation associated [Source:HGNC Symbol;Acc:HGNC:13940]" | 4641 | 0.101 | -0.0600 | No |
| 111 | PTBP1 | polypyrimidine tract binding protein 1 [Source:HGNC Symbol;Acc:HGNC:9583] | 4650 | 0.100 | -0.0599 | No |
| 112 | GRSF1 | G-rich RNA sequence binding factor 1 [Source:HGNC Symbol;Acc:HGNC:4610] | 4660 | 0.099 | -0.0598 | No |
| 113 | RNGTT | RNA guanylyltransferase and 5'-phosphatase [Source:HGNC Symbol;Acc:HGNC:10073] | 4737 | 0.092 | -0.0652 | No |
| 114 | DDX47 | DEAD-box helicase 47 [Source:HGNC Symbol;Acc:HGNC:18682] | 4789 | 0.088 | -0.0686 | No |
| 115 | ZC3H14 | zinc finger CCCH-type containing 14 [Source:HGNC Symbol;Acc:HGNC:20509] | 4896 | 0.080 | -0.0764 | No |
| 116 | DDX41 | DEAD-box helicase 41 [Source:HGNC Symbol;Acc:HGNC:18674] | 5004 | 0.073 | -0.0844 | No |
| 117 | NRDE2 | "NRDE-2, necessary for RNA interference, domain containing [Source:HGNC Symbol;Acc:HGNC:20186]" | 5019 | 0.071 | -0.0850 | No |
| 118 | PRPF4 | pre-mRNA processing factor 4 [Source:HGNC Symbol;Acc:HGNC:17349] | 5076 | 0.067 | -0.0889 | No |
| 119 | IK | IK cytokine [Source:HGNC Symbol;Acc:HGNC:5958] | 5225 | 0.054 | -0.1003 | No |
| 120 | CDC40 | cell division cycle 40 [Source:HGNC Symbol;Acc:HGNC:17350] | 5228 | 0.054 | -0.1001 | No |
| 121 | SNRPB2 | small nuclear ribonucleoprotein polypeptide B2 [Source:HGNC Symbol;Acc:HGNC:11155] | 5266 | 0.050 | -0.1026 | No |
| 122 | PRPF8 | pre-mRNA processing factor 8 [Source:HGNC Symbol;Acc:HGNC:17340] | 5287 | 0.049 | -0.1039 | No |
| 123 | STRAP | serine/threonine kinase receptor associated protein [Source:HGNC Symbol;Acc:HGNC:30796] | 5375 | 0.042 | -0.1105 | No |
| 124 | CDC5L | cell division cycle 5 like [Source:HGNC Symbol;Acc:HGNC:1743] | 5460 | 0.036 | -0.1169 | No |
| 125 | SNW1 | SNW domain containing 1 [Source:HGNC Symbol;Acc:HGNC:16696] | 5472 | 0.035 | -0.1175 | No |
| 126 | HNRNPA0 | heterogeneous nuclear ribonucleoprotein A0 [Source:HGNC Symbol;Acc:HGNC:5030] | 5485 | 0.033 | -0.1182 | No |
| 127 | DHX8 | DEAH-box helicase 8 [Source:HGNC Symbol;Acc:HGNC:2749] | 5531 | 0.030 | -0.1216 | No |
| 128 | SNRPE | small nuclear ribonucleoprotein polypeptide E [Source:HGNC Symbol;Acc:HGNC:11161] | 5551 | 0.029 | -0.1229 | No |
| 129 | CDC73 | cell division cycle 73 [Source:HGNC Symbol;Acc:HGNC:16783] | 5604 | 0.023 | -0.1269 | No |
| 130 | ERN1 | endoplasmic reticulum to nucleus signaling 1 [Source:HGNC Symbol;Acc:HGNC:3449] | 5658 | 0.019 | -0.1309 | No |
| 131 | SART1 | "spliceosome associated factor 1, recruiter of U4/U6.U5 tri-snRNP [Source:HGNC Symbol;Acc:HGNC:10538]" | 5690 | 0.017 | -0.1333 | No |
| 132 | DDX46 | DEAD-box helicase 46 [Source:HGNC Symbol;Acc:HGNC:18681] | 5744 | 0.012 | -0.1374 | No |
| 133 | HNRNPH3 | heterogeneous nuclear ribonucleoprotein H3 [Source:HGNC Symbol;Acc:HGNC:5043] | 5745 | 0.012 | -0.1373 | No |
| 134 | PQBP1 | polyglutamine binding protein 1 [Source:HGNC Symbol;Acc:HGNC:9330] | 5827 | 0.007 | -0.1437 | No |
| 135 | AHCYL1 | adenosylhomocysteinase like 1 [Source:HGNC Symbol;Acc:HGNC:344] | 5837 | 0.006 | -0.1444 | No |
| 136 | SF3B2 | splicing factor 3b subunit 2 [Source:HGNC Symbol;Acc:HGNC:10769] | 5895 | 0.002 | -0.1489 | No |
| 137 | PAF1 | "PAF1 homolog, Paf1/RNA polymerase II complex component [Source:HGNC Symbol;Acc:HGNC:25459]" | 5901 | 0.002 | -0.1493 | No |
| 138 | FASTKD5 | FAST kinase domains 5 [Source:HGNC Symbol;Acc:HGNC:25790] | 5982 | -0.003 | -0.1557 | No |
| 139 | SF3A2 | splicing factor 3a subunit 2 [Source:HGNC Symbol;Acc:HGNC:10766] | 5999 | -0.004 | -0.1569 | No |
| 140 | RBMXL2 | RBMX like 2 [Source:HGNC Symbol;Acc:HGNC:17886] | 6034 | -0.007 | -0.1596 | No |
| 141 | RBM42 | RNA binding motif protein 42 [Source:HGNC Symbol;Acc:HGNC:28117] | 6059 | -0.009 | -0.1614 | No |
| 142 | RBFOX1 | RNA binding fox-1 homolog 1 [Source:HGNC Symbol;Acc:HGNC:18222] | 6152 | -0.016 | -0.1687 | No |
| 143 | WDR33 | WD repeat domain 33 [Source:HGNC Symbol;Acc:HGNC:25651] | 6249 | -0.024 | -0.1761 | No |
| 144 | RPRD1A | regulation of nuclear pre-mRNA domain containing 1A [Source:HGNC Symbol;Acc:HGNC:25560] | 6294 | -0.027 | -0.1794 | No |
| 145 | TSEN2 | tRNA splicing endonuclease subunit 2 [Source:HGNC Symbol;Acc:HGNC:28422] | 6331 | -0.031 | -0.1821 | No |
| 146 | SF3B3 | splicing factor 3b subunit 3 [Source:HGNC Symbol;Acc:HGNC:10770] | 6372 | -0.033 | -0.1850 | No |
| 147 | AAR2 | AAR2 splicing factor [Source:HGNC Symbol;Acc:HGNC:15886] | 6401 | -0.035 | -0.1870 | No |
| 148 | CTR9 | "CTR9 homolog, Paf1/RNA polymerase II complex component [Source:HGNC Symbol;Acc:HGNC:16850]" | 6430 | -0.037 | -0.1889 | No |
| 149 | CPEB1 | cytoplasmic polyadenylation element binding protein 1 [Source:HGNC Symbol;Acc:HGNC:21744] | 6435 | -0.038 | -0.1890 | No |
| 150 | SNIP1 | Smad nuclear interacting protein 1 [Source:HGNC Symbol;Acc:HGNC:30587] | 6466 | -0.040 | -0.1910 | No |
| 151 | WTAP | WT1 associated protein [Source:HGNC Symbol;Acc:HGNC:16846] | 6598 | -0.049 | -0.2011 | No |
| 152 | CPSF1 | cleavage and polyadenylation specific factor 1 [Source:HGNC Symbol;Acc:HGNC:2324] | 6629 | -0.052 | -0.2031 | No |
| 153 | RBM10 | RNA binding motif protein 10 [Source:HGNC Symbol;Acc:HGNC:9896] | 6647 | -0.053 | -0.2041 | No |
| 154 | WBP4 | WW domain binding protein 4 [Source:HGNC Symbol;Acc:HGNC:12739] | 6678 | -0.054 | -0.2060 | No |
| 155 | RNF40 | ring finger protein 40 [Source:HGNC Symbol;Acc:HGNC:16867] | 6681 | -0.054 | -0.2058 | No |
| 156 | METTL3 | "methyltransferase 3, N6-adenosine-methyltransferase complex catalytic subunit [Source:HGNC Symbol;Acc:HGNC:17563]" | 6685 | -0.055 | -0.2056 | No |
| 157 | SF3B4 | splicing factor 3b subunit 4 [Source:HGNC Symbol;Acc:HGNC:10771] | 6777 | -0.061 | -0.2124 | No |
| 158 | PRDX6 | peroxiredoxin 6 [Source:HGNC Symbol;Acc:HGNC:16753] | 6783 | -0.061 | -0.2123 | No |
| 159 | LSM8 | "LSM8 homolog, U6 small nuclear RNA associated [Source:HGNC Symbol;Acc:HGNC:20471]" | 6828 | -0.065 | -0.2153 | No |
| 160 | RBM15B | RNA binding motif protein 15B [Source:HGNC Symbol;Acc:HGNC:24303] | 6867 | -0.069 | -0.2178 | No |
| 161 | SNRNP200 | small nuclear ribonucleoprotein U5 subunit 200 [Source:HGNC Symbol;Acc:HGNC:30859] | 7249 | -0.099 | -0.2475 | No |
| 162 | SUGP1 | SURP and G-patch domain containing 1 [Source:HGNC Symbol;Acc:HGNC:18643] | 7267 | -0.100 | -0.2481 | No |
| 163 | PAPOLG | poly(A) polymerase gamma [Source:HGNC Symbol;Acc:HGNC:14982] | 7268 | -0.100 | -0.2473 | No |
| 164 | PRKRIP1 | PRKR interacting protein 1 [Source:HGNC Symbol;Acc:HGNC:21894] | 7282 | -0.101 | -0.2476 | No |
| 165 | SMU1 | SMU1 DNA replication regulator and spliceosomal factor [Source:HGNC Symbol;Acc:HGNC:18247] | 7299 | -0.102 | -0.2481 | No |
| 166 | WBP11 | WW domain binding protein 11 [Source:HGNC Symbol;Acc:HGNC:16461] | 7312 | -0.103 | -0.2482 | No |
| 167 | NOVA1 | NOVA alternative splicing regulator 1 [Source:HGNC Symbol;Acc:HGNC:7886] | 7394 | -0.110 | -0.2538 | No |
| 168 | HNRNPC | heterogeneous nuclear ribonucleoprotein C [Source:HGNC Symbol;Acc:HGNC:5035] | 7437 | -0.114 | -0.2563 | No |
| 169 | CWC25 | CWC25 spliceosome associated protein homolog [Source:HGNC Symbol;Acc:HGNC:25989] | 7482 | -0.117 | -0.2589 | No |
| 170 | ZNF473 | zinc finger protein 473 [Source:HGNC Symbol;Acc:HGNC:23239] | 7497 | -0.118 | -0.2591 | No |
| 171 | MAGOHB | "mago homolog B, exon junction complex subunit [Source:HGNC Symbol;Acc:HGNC:25504]" | 7506 | -0.119 | -0.2589 | No |
| 172 | NOL3 | nucleolar protein 3 [Source:HGNC Symbol;Acc:HGNC:7869] | 7545 | -0.122 | -0.2610 | No |
| 173 | ERN2 | endoplasmic reticulum to nucleus signaling 2 [Source:HGNC Symbol;Acc:HGNC:16942] | 7565 | -0.124 | -0.2615 | No |
| 174 | RBMX2 | RNA binding motif protein X-linked 2 [Source:HGNC Symbol;Acc:HGNC:24282] | 7614 | -0.128 | -0.2644 | No |
| 175 | EIF4A3 | eukaryotic translation initiation factor 4A3 [Source:HGNC Symbol;Acc:HGNC:18683] | 7646 | -0.130 | -0.2658 | No |
| 176 | AQR | aquarius intron-binding spliceosomal factor [Source:HGNC Symbol;Acc:HGNC:29513] | 7654 | -0.131 | -0.2654 | No |
| 177 | DYRK1A | dual specificity tyrosine phosphorylation regulated kinase 1A [Source:HGNC Symbol;Acc:HGNC:3091] | 7667 | -0.132 | -0.2653 | No |
| 178 | POLR2D | RNA polymerase II subunit D [Source:HGNC Symbol;Acc:HGNC:9191] | 7726 | -0.136 | -0.2689 | No |
| 179 | TTF2 | transcription termination factor 2 [Source:HGNC Symbol;Acc:HGNC:12398] | 7764 | -0.139 | -0.2708 | No |
| 180 | TGS1 | trimethylguanosine synthase 1 [Source:HGNC Symbol;Acc:HGNC:17843] | 7791 | -0.141 | -0.2718 | No |
| 181 | APP | amyloid beta precursor protein [Source:HGNC Symbol;Acc:HGNC:620] | 7835 | -0.143 | -0.2741 | No |
| 182 | MYOD1 | myogenic differentiation 1 [Source:HGNC Symbol;Acc:HGNC:7611] | 7839 | -0.143 | -0.2733 | No |
| 183 | TARDBP | TAR DNA binding protein [Source:HGNC Symbol;Acc:HGNC:11571] | 7864 | -0.146 | -0.2741 | No |
| 184 | CPSF6 | cleavage and polyadenylation specific factor 6 [Source:HGNC Symbol;Acc:HGNC:13871] | 7885 | -0.147 | -0.2745 | No |
| 185 | PRPF31 | pre-mRNA processing factor 31 [Source:HGNC Symbol;Acc:HGNC:15446] | 7898 | -0.147 | -0.2744 | No |
| 186 | PRMT5 | protein arginine methyltransferase 5 [Source:HGNC Symbol;Acc:HGNC:10894] | 7904 | -0.148 | -0.2736 | No |
| 187 | PAPOLB | poly(A) polymerase beta [Source:HGNC Symbol;Acc:HGNC:15970] | 7906 | -0.148 | -0.2726 | No |
| 188 | FMR1 | FMRP translational regulator 1 [Source:HGNC Symbol;Acc:HGNC:3775] | 7944 | -0.151 | -0.2744 | No |
| 189 | CMTR1 | cap methyltransferase 1 [Source:HGNC Symbol;Acc:HGNC:21077] | 7951 | -0.151 | -0.2737 | No |
| 190 | CPEB3 | cytoplasmic polyadenylation element binding protein 3 [Source:HGNC Symbol;Acc:HGNC:21746] | 8060 | -0.160 | -0.2811 | No |
| 191 | HNRNPM | heterogeneous nuclear ribonucleoprotein M [Source:HGNC Symbol;Acc:HGNC:5046] | 8127 | -0.165 | -0.2851 | No |
| 192 | RNPS1 | RNA binding protein with serine rich domain 1 [Source:HGNC Symbol;Acc:HGNC:10080] | 8275 | -0.177 | -0.2955 | No |
| 193 | KIN | Kin17 DNA and RNA binding protein [Source:HGNC Symbol;Acc:HGNC:6327] | 8280 | -0.177 | -0.2944 | No |
| 194 | CSDC2 | cold shock domain containing C2 [Source:HGNC Symbol;Acc:HGNC:30359] | 8294 | -0.178 | -0.2941 | No |
| 195 | PDE12 | phosphodiesterase 12 [Source:HGNC Symbol;Acc:HGNC:25386] | 8306 | -0.178 | -0.2936 | No |
| 196 | SETX | senataxin [Source:HGNC Symbol;Acc:HGNC:445] | 8398 | -0.185 | -0.2994 | No |
| 197 | NCBP2 | nuclear cap binding protein subunit 2 [Source:HGNC Symbol;Acc:HGNC:7659] | 8478 | -0.192 | -0.3043 | No |
| 198 | THOC5 | THO complex 5 [Source:HGNC Symbol;Acc:HGNC:19074] | 8549 | -0.198 | -0.3083 | No |
| 199 | HNRNPA2B1 | heterogeneous nuclear ribonucleoprotein A2/B1 [Source:HGNC Symbol;Acc:HGNC:5033] | 8576 | -0.200 | -0.3089 | No |
| 200 | HNRNPA3 | heterogeneous nuclear ribonucleoprotein A3 [Source:HGNC Symbol;Acc:HGNC:24941] | 8603 | -0.201 | -0.3094 | No |
| 201 | KDM1A | lysine demethylase 1A [Source:HGNC Symbol;Acc:HGNC:29079] | 8608 | -0.202 | -0.3082 | No |
| 202 | RBM8A | RNA binding motif protein 8A [Source:HGNC Symbol;Acc:HGNC:9905] | 8662 | -0.205 | -0.3108 | No |
| 203 | RBM41 | RNA binding motif protein 41 [Source:HGNC Symbol;Acc:HGNC:25617] | 8705 | -0.210 | -0.3126 | No |
| 204 | SUPT6H | "SPT6 homolog, histone chaperone and transcription elongation factor [Source:HGNC Symbol;Acc:HGNC:11470]" | 8752 | -0.213 | -0.3146 | No |
| 205 | XAB2 | XPA binding protein 2 [Source:HGNC Symbol;Acc:HGNC:14089] | 8778 | -0.215 | -0.3150 | No |
| 206 | RBM3 | RNA binding motif protein 3 [Source:HGNC Symbol;Acc:HGNC:9900] | 8859 | -0.222 | -0.3196 | No |
| 207 | AFF2 | AF4/FMR2 family member 2 [Source:HGNC Symbol;Acc:HGNC:3776] | 8869 | -0.222 | -0.3187 | No |
| 208 | HNRNPL | heterogeneous nuclear ribonucleoprotein L [Source:HGNC Symbol;Acc:HGNC:5045] | 8892 | -0.224 | -0.3187 | No |
| 209 | RRP1B | ribosomal RNA processing 1B [Source:HGNC Symbol;Acc:HGNC:23818] | 8909 | -0.225 | -0.3182 | No |
| 210 | RBMX | RNA binding motif protein X-linked [Source:HGNC Symbol;Acc:HGNC:9910] | 8938 | -0.227 | -0.3187 | No |
| 211 | RBMY1A1 | RNA binding motif protein Y-linked family 1 member A1 [Source:HGNC Symbol;Acc:HGNC:9912] | 9014 | -0.233 | -0.3229 | No |
| 212 | SNRPG | small nuclear ribonucleoprotein polypeptide G [Source:HGNC Symbol;Acc:HGNC:11163] | 9067 | -0.237 | -0.3253 | No |
| 213 | PPWD1 | peptidylprolyl isomerase domain and WD repeat containing 1 [Source:HGNC Symbol;Acc:HGNC:28954] | 9069 | -0.237 | -0.3235 | No |
| 214 | SNRPB | small nuclear ribonucleoprotein polypeptides B and B1 [Source:HGNC Symbol;Acc:HGNC:11153] | 9085 | -0.239 | -0.3229 | No |
| 215 | TFIP11 | tuftelin interacting protein 11 [Source:HGNC Symbol;Acc:HGNC:17165] | 9098 | -0.239 | -0.3220 | No |
| 216 | USP4 | ubiquitin specific peptidase 4 [Source:HGNC Symbol;Acc:HGNC:12627] | 9130 | -0.242 | -0.3226 | No |
| 217 | HSPA8 | heat shock protein family A (Hsp70) member 8 [Source:HGNC Symbol;Acc:HGNC:5241] | 9140 | -0.243 | -0.3215 | No |
| 218 | NCBP1 | nuclear cap binding protein subunit 1 [Source:HGNC Symbol;Acc:HGNC:7658] | 9149 | -0.244 | -0.3202 | No |
| 219 | ZPR1 | ZPR1 zinc finger [Source:HGNC Symbol;Acc:HGNC:13051] | 9151 | -0.244 | -0.3184 | No |
| 220 | ADAR | adenosine deaminase RNA specific [Source:HGNC Symbol;Acc:HGNC:225] | 9195 | -0.247 | -0.3200 | No |
| 221 | PRPF4B | pre-mRNA processing factor 4B [Source:HGNC Symbol;Acc:HGNC:17346] | 9210 | -0.249 | -0.3192 | No |
| 222 | CDK12 | cyclin dependent kinase 12 [Source:HGNC Symbol;Acc:HGNC:24224] | 9225 | -0.250 | -0.3184 | No |
| 223 | TRA2B | transformer 2 beta homolog [Source:HGNC Symbol;Acc:HGNC:10781] | 9243 | -0.251 | -0.3178 | No |
| 224 | SNRNP40 | small nuclear ribonucleoprotein U5 subunit 40 [Source:HGNC Symbol;Acc:HGNC:30857] | 9295 | -0.256 | -0.3199 | No |
| 225 | PAXBP1 | PAX3 and PAX7 binding protein 1 [Source:HGNC Symbol;Acc:HGNC:13579] | 9333 | -0.258 | -0.3209 | No |
| 226 | NONO | non-POU domain containing octamer binding [Source:HGNC Symbol;Acc:HGNC:7871] | 9334 | -0.258 | -0.3189 | No |
| 227 | HNRNPR | heterogeneous nuclear ribonucleoprotein R [Source:HGNC Symbol;Acc:HGNC:5047] | 9430 | -0.267 | -0.3244 | No |
| 228 | GEMIN7 | gem nuclear organelle associated protein 7 [Source:HGNC Symbol;Acc:HGNC:20045] | 9441 | -0.268 | -0.3232 | No |
| 229 | LGALS3 | galectin 3 [Source:HGNC Symbol;Acc:HGNC:6563] | 9508 | -0.274 | -0.3263 | No |
| 230 | ZC3H13 | zinc finger CCCH-type containing 13 [Source:HGNC Symbol;Acc:HGNC:20368] | 9558 | -0.279 | -0.3281 | No |
| 231 | SNRPD1 | small nuclear ribonucleoprotein D1 polypeptide [Source:HGNC Symbol;Acc:HGNC:11158] | 9579 | -0.281 | -0.3275 | No |
| 232 | YBX1 | Y-box binding protein 1 [Source:HGNC Symbol;Acc:HGNC:8014] | 9623 | -0.285 | -0.3288 | No |
| 233 | ZCCHC8 | zinc finger CCHC-type containing 8 [Source:HGNC Symbol;Acc:HGNC:25265] | 9624 | -0.285 | -0.3266 | No |
| 234 | SMNDC1 | survival motor neuron domain containing 1 [Source:HGNC Symbol;Acc:HGNC:16900] | 9638 | -0.287 | -0.3254 | No |
| 235 | CIR1 | "corepressor interacting with RBPJ, CIR1 [Source:HGNC Symbol;Acc:HGNC:24217]" | 9640 | -0.287 | -0.3233 | No |
| 236 | RBM14 | RNA binding motif protein 14 [Source:HGNC Symbol;Acc:HGNC:14219] | 9663 | -0.288 | -0.3229 | No |
| 237 | SFPQ | splicing factor proline and glutamine rich [Source:HGNC Symbol;Acc:HGNC:10774] | 9747 | -0.296 | -0.3272 | No |
| 238 | PAPOLA | poly(A) polymerase alpha [Source:HGNC Symbol;Acc:HGNC:14981] | 9786 | -0.300 | -0.3279 | No |
| 239 | TBRG4 | transforming growth factor beta regulator 4 [Source:HGNC Symbol;Acc:HGNC:17443] | 9818 | -0.302 | -0.3281 | No |
| 240 | HNRNPU | heterogeneous nuclear ribonucleoprotein U [Source:HGNC Symbol;Acc:HGNC:5048] | 9847 | -0.306 | -0.3280 | No |
| 241 | GCFC2 | GC-rich sequence DNA-binding factor 2 [Source:HGNC Symbol;Acc:HGNC:1317] | 9908 | -0.312 | -0.3304 | No |
| 242 | RNASEL | ribonuclease L [Source:HGNC Symbol;Acc:HGNC:10050] | 9965 | -0.317 | -0.3324 | No |
| 243 | KHDC4 | "KH domain containing 4, pre-mRNA splicing factor [Source:HGNC Symbol;Acc:HGNC:29145]" | 9997 | -0.321 | -0.3324 | No |
| 244 | TENT4A | terminal nucleotidyltransferase 4A [Source:HGNC Symbol;Acc:HGNC:16705] | 10003 | -0.322 | -0.3303 | No |
| 245 | GEMIN4 | gem nuclear organelle associated protein 4 [Source:HGNC Symbol;Acc:HGNC:15717] | 10075 | -0.328 | -0.3335 | No |
| 246 | FIP1L1 | factor interacting with PAPOLA and CPSF1 [Source:HGNC Symbol;Acc:HGNC:19124] | 10187 | -0.339 | -0.3397 | Yes |
| 247 | C9orf78 | chromosome 9 open reading frame 78 [Source:HGNC Symbol;Acc:HGNC:24932] | 10210 | -0.342 | -0.3389 | Yes |
| 248 | DHX35 | DEAH-box helicase 35 [Source:HGNC Symbol;Acc:HGNC:15861] | 10215 | -0.342 | -0.3366 | Yes |
| 249 | PTBP3 | polypyrimidine tract binding protein 3 [Source:HGNC Symbol;Acc:HGNC:10253] | 10259 | -0.347 | -0.3373 | Yes |
| 250 | SRSF1 | serine and arginine rich splicing factor 1 [Source:HGNC Symbol;Acc:HGNC:10780] | 10272 | -0.348 | -0.3356 | Yes |
| 251 | SRSF3 | serine and arginine rich splicing factor 3 [Source:HGNC Symbol;Acc:HGNC:10785] | 10280 | -0.349 | -0.3335 | Yes |
| 252 | SRSF5 | serine and arginine rich splicing factor 5 [Source:HGNC Symbol;Acc:HGNC:10787] | 10282 | -0.349 | -0.3309 | Yes |
| 253 | ADARB1 | adenosine deaminase RNA specific B1 [Source:HGNC Symbol;Acc:HGNC:226] | 10295 | -0.350 | -0.3292 | Yes |
| 254 | SF3A3 | splicing factor 3a subunit 3 [Source:HGNC Symbol;Acc:HGNC:10767] | 10311 | -0.352 | -0.3277 | Yes |
| 255 | LUC7L2 | "LUC7 like 2, pre-mRNA splicing factor [Source:HGNC Symbol;Acc:HGNC:21608]" | 10334 | -0.354 | -0.3267 | Yes |
| 256 | RBM22 | RNA binding motif protein 22 [Source:HGNC Symbol;Acc:HGNC:25503] | 10345 | -0.355 | -0.3248 | Yes |
| 257 | SCAF11 | SR-related CTD associated factor 11 [Source:HGNC Symbol;Acc:HGNC:10784] | 10375 | -0.358 | -0.3243 | Yes |
| 258 | ELAVL4 | ELAV like RNA binding protein 4 [Source:HGNC Symbol;Acc:HGNC:3315] | 10412 | -0.362 | -0.3244 | Yes |
| 259 | CSTF2 | cleavage stimulation factor subunit 2 [Source:HGNC Symbol;Acc:HGNC:2484] | 10438 | -0.366 | -0.3236 | Yes |
| 260 | HABP4 | hyaluronan binding protein 4 [Source:HGNC Symbol;Acc:HGNC:17062] | 10485 | -0.371 | -0.3244 | Yes |
| 261 | DHX15 | DEAH-box helicase 15 [Source:HGNC Symbol;Acc:HGNC:2738] | 10498 | -0.372 | -0.3225 | Yes |
| 262 | RBM15 | RNA binding motif protein 15 [Source:HGNC Symbol;Acc:HGNC:14959] | 10499 | -0.373 | -0.3197 | Yes |
| 263 | PPARGC1A | PPARG coactivator 1 alpha [Source:HGNC Symbol;Acc:HGNC:9237] | 10504 | -0.374 | -0.3171 | Yes |
| 264 | SRSF2 | serine and arginine rich splicing factor 2 [Source:HGNC Symbol;Acc:HGNC:10783] | 10507 | -0.374 | -0.3144 | Yes |
| 265 | PCF11 | PCF11 cleavage and polyadenylation factor subunit [Source:HGNC Symbol;Acc:HGNC:30097] | 10521 | -0.376 | -0.3126 | Yes |
| 266 | SRPK2 | SRSF protein kinase 2 [Source:HGNC Symbol;Acc:HGNC:11306] | 10522 | -0.376 | -0.3097 | Yes |
| 267 | MBNL1 | muscleblind like splicing regulator 1 [Source:HGNC Symbol;Acc:HGNC:6923] | 10557 | -0.379 | -0.3095 | Yes |
| 268 | PRKACA | protein kinase cAMP-activated catalytic subunit alpha [Source:HGNC Symbol;Acc:HGNC:9380] | 10611 | -0.384 | -0.3108 | Yes |
| 269 | DHX9 | DExH-box helicase 9 [Source:HGNC Symbol;Acc:HGNC:2750] | 10627 | -0.386 | -0.3090 | Yes |
| 270 | LUC7L3 | LUC7 like 3 pre-mRNA splicing factor [Source:HGNC Symbol;Acc:HGNC:24309] | 10647 | -0.388 | -0.3075 | Yes |
| 271 | SRSF6 | serine and arginine rich splicing factor 6 [Source:HGNC Symbol;Acc:HGNC:10788] | 10659 | -0.389 | -0.3054 | Yes |
| 272 | CDK13 | cyclin dependent kinase 13 [Source:HGNC Symbol;Acc:HGNC:1733] | 10675 | -0.390 | -0.3036 | Yes |
| 273 | YTHDC1 | YTH domain containing 1 [Source:HGNC Symbol;Acc:HGNC:30626] | 10687 | -0.391 | -0.3015 | Yes |
| 274 | TUT1 | "terminal uridylyl transferase 1, U6 snRNA-specific [Source:HGNC Symbol;Acc:HGNC:26184]" | 10699 | -0.393 | -0.2994 | Yes |
| 275 | U2AF1 | U2 small nuclear RNA auxiliary factor 1 [Source:HGNC Symbol;Acc:HGNC:12453] | 10762 | -0.400 | -0.3012 | Yes |
| 276 | RBM25 | RNA binding motif protein 25 [Source:HGNC Symbol;Acc:HGNC:23244] | 10800 | -0.405 | -0.3011 | Yes |
| 277 | CELF2 | CUGBP Elav-like family member 2 [Source:HGNC Symbol;Acc:HGNC:2550] | 10832 | -0.409 | -0.3004 | Yes |
| 278 | RBM5 | RNA binding motif protein 5 [Source:HGNC Symbol;Acc:HGNC:9902] | 10849 | -0.410 | -0.2985 | Yes |
| 279 | TIA1 | TIA1 cytotoxic granule associated RNA binding protein [Source:HGNC Symbol;Acc:HGNC:11802] | 10855 | -0.411 | -0.2958 | Yes |
| 280 | NUP98 | nucleoporin 98 and 96 precursor [Source:HGNC Symbol;Acc:HGNC:8068] | 10875 | -0.413 | -0.2941 | Yes |
| 281 | SRSF10 | serine and arginine rich splicing factor 10 [Source:HGNC Symbol;Acc:HGNC:16713] | 10926 | -0.422 | -0.2949 | Yes |
| 282 | SRSF7 | serine and arginine rich splicing factor 7 [Source:HGNC Symbol;Acc:HGNC:10789] | 10930 | -0.422 | -0.2919 | Yes |
| 283 | SF3B1 | splicing factor 3b subunit 1 [Source:HGNC Symbol;Acc:HGNC:10768] | 10948 | -0.424 | -0.2900 | Yes |
| 284 | RBM19 | RNA binding motif protein 19 [Source:HGNC Symbol;Acc:HGNC:29098] | 11030 | -0.434 | -0.2931 | Yes |
| 285 | CDK9 | cyclin dependent kinase 9 [Source:HGNC Symbol;Acc:HGNC:1780] | 11071 | -0.441 | -0.2929 | Yes |
| 286 | PRPF38B | pre-mRNA processing factor 38B [Source:HGNC Symbol;Acc:HGNC:25512] | 11147 | -0.450 | -0.2954 | Yes |
| 287 | SYNCRIP | synaptotagmin binding cytoplasmic RNA interacting protein [Source:HGNC Symbol;Acc:HGNC:16918] | 11182 | -0.454 | -0.2947 | Yes |
| 288 | HSF1 | heat shock transcription factor 1 [Source:HGNC Symbol;Acc:HGNC:5224] | 11189 | -0.456 | -0.2917 | Yes |
| 289 | SNRPA1 | small nuclear ribonucleoprotein polypeptide A' [Source:HGNC Symbol;Acc:HGNC:11152] | 11218 | -0.459 | -0.2904 | Yes |
| 290 | PRPF19 | pre-mRNA processing factor 19 [Source:HGNC Symbol;Acc:HGNC:17896] | 11269 | -0.467 | -0.2908 | Yes |
| 291 | KHSRP | KH-type splicing regulatory protein [Source:HGNC Symbol;Acc:HGNC:6316] | 11285 | -0.469 | -0.2884 | Yes |
| 292 | SRRM1 | serine and arginine repetitive matrix 1 [Source:HGNC Symbol;Acc:HGNC:16638] | 11302 | -0.471 | -0.2860 | Yes |
| 293 | HNRNPF | heterogeneous nuclear ribonucleoprotein F [Source:HGNC Symbol;Acc:HGNC:5039] | 11339 | -0.476 | -0.2852 | Yes |
| 294 | METTL16 | "methyltransferase 16, N6-methyladenosine [Source:HGNC Symbol;Acc:HGNC:28484]" | 11346 | -0.478 | -0.2820 | Yes |
| 295 | DDX39A | DExD-box helicase 39A [Source:HGNC Symbol;Acc:HGNC:17821] | 11359 | -0.480 | -0.2793 | Yes |
| 296 | TXNL4B | thioredoxin like 4B [Source:HGNC Symbol;Acc:HGNC:26041] | 11370 | -0.482 | -0.2764 | Yes |
| 297 | RNMT | RNA guanine-7 methyltransferase [Source:HGNC Symbol;Acc:HGNC:10075] | 11392 | -0.485 | -0.2744 | Yes |
| 298 | RBM39 | RNA binding motif protein 39 [Source:HGNC Symbol;Acc:HGNC:15923] | 11434 | -0.493 | -0.2738 | Yes |
| 299 | NCBP3 | nuclear cap binding subunit 3 [Source:HGNC Symbol;Acc:HGNC:24612] | 11502 | -0.501 | -0.2753 | Yes |
| 300 | CELF3 | CUGBP Elav-like family member 3 [Source:HGNC Symbol;Acc:HGNC:11967] | 11510 | -0.502 | -0.2721 | Yes |
| 301 | ZC3H3 | zinc finger CCCH-type containing 3 [Source:HGNC Symbol;Acc:HGNC:28972] | 11571 | -0.511 | -0.2729 | Yes |
| 302 | SLTM | SAFB like transcription modulator [Source:HGNC Symbol;Acc:HGNC:20709] | 11593 | -0.514 | -0.2706 | Yes |
| 303 | ZBTB7A | zinc finger and BTB domain containing 7A [Source:HGNC Symbol;Acc:HGNC:18078] | 11600 | -0.516 | -0.2672 | Yes |
| 304 | SRRT | "serrate, RNA effector molecule [Source:HGNC Symbol;Acc:HGNC:24101]" | 11626 | -0.521 | -0.2652 | Yes |
| 305 | LUC7L | LUC7 like [Source:HGNC Symbol;Acc:HGNC:6723] | 11627 | -0.521 | -0.2612 | Yes |
| 306 | CASC3 | CASC3 exon junction complex subunit [Source:HGNC Symbol;Acc:HGNC:17040] | 11630 | -0.522 | -0.2573 | Yes |
| 307 | SF1 | splicing factor 1 [Source:HGNC Symbol;Acc:HGNC:12950] | 11664 | -0.527 | -0.2559 | Yes |
| 308 | DDX17 | DEAD-box helicase 17 [Source:HGNC Symbol;Acc:HGNC:2740] | 11716 | -0.535 | -0.2559 | Yes |
| 309 | SUPT5H | "SPT5 homolog, DSIF elongation factor subunit [Source:HGNC Symbol;Acc:HGNC:11469]" | 11735 | -0.539 | -0.2532 | Yes |
| 310 | SYMPK | symplekin [Source:HGNC Symbol;Acc:HGNC:22935] | 11757 | -0.542 | -0.2507 | Yes |
| 311 | PDCD11 | programmed cell death 11 [Source:HGNC Symbol;Acc:HGNC:13408] | 11847 | -0.559 | -0.2535 | Yes |
| 312 | PRPF39 | pre-mRNA processing factor 39 [Source:HGNC Symbol;Acc:HGNC:20314] | 11854 | -0.560 | -0.2497 | Yes |
| 313 | DHX38 | DEAH-box helicase 38 [Source:HGNC Symbol;Acc:HGNC:17211] | 11860 | -0.562 | -0.2457 | Yes |
| 314 | SRPK1 | SRSF protein kinase 1 [Source:HGNC Symbol;Acc:HGNC:11305] | 11889 | -0.569 | -0.2436 | Yes |
| 315 | THOC6 | THO complex 6 [Source:HGNC Symbol;Acc:HGNC:28369] | 11918 | -0.576 | -0.2414 | Yes |
| 316 | TCERG1 | transcription elongation regulator 1 [Source:HGNC Symbol;Acc:HGNC:15630] | 11950 | -0.583 | -0.2394 | Yes |
| 317 | CDK11B | cyclin dependent kinase 11B [Source:HGNC Symbol;Acc:HGNC:1729] | 11952 | -0.584 | -0.2350 | Yes |
| 318 | PNN | "pinin, desmosome associated protein [Source:HGNC Symbol;Acc:HGNC:9162]" | 11954 | -0.584 | -0.2306 | Yes |
| 319 | NOVA2 | NOVA alternative splicing regulator 2 [Source:HGNC Symbol;Acc:HGNC:7887] | 12006 | -0.594 | -0.2301 | Yes |
| 320 | RBM38 | RNA binding motif protein 38 [Source:HGNC Symbol;Acc:HGNC:15818] | 12026 | -0.599 | -0.2270 | Yes |
| 321 | ACIN1 | apoptotic chromatin condensation inducer 1 [Source:HGNC Symbol;Acc:HGNC:17066] | 12059 | -0.609 | -0.2249 | Yes |
| 322 | SNRNP70 | small nuclear ribonucleoprotein U1 subunit 70 [Source:HGNC Symbol;Acc:HGNC:11150] | 12073 | -0.613 | -0.2212 | Yes |
| 323 | SMN1 | "survival of motor neuron 1, telomeric [Source:HGNC Symbol;Acc:HGNC:11117]" | 12102 | -0.621 | -0.2187 | Yes |
| 324 | CPSF4 | cleavage and polyadenylation specific factor 4 [Source:HGNC Symbol;Acc:HGNC:2327] | 12119 | -0.624 | -0.2152 | Yes |
| 325 | RBM26 | RNA binding motif protein 26 [Source:HGNC Symbol;Acc:HGNC:20327] | 12139 | -0.628 | -0.2119 | Yes |
| 326 | SART3 | "spliceosome associated factor 3, U4/U6 recycling protein [Source:HGNC Symbol;Acc:HGNC:16860]" | 12161 | -0.633 | -0.2087 | Yes |
| 327 | RSRC1 | arginine and serine rich coiled-coil 1 [Source:HGNC Symbol;Acc:HGNC:24152] | 12165 | -0.635 | -0.2041 | Yes |
| 328 | PTBP2 | polypyrimidine tract binding protein 2 [Source:HGNC Symbol;Acc:HGNC:17662] | 12211 | -0.649 | -0.2027 | Yes |
| 329 | FXR2 | FMR1 autosomal homolog 2 [Source:HGNC Symbol;Acc:HGNC:4024] | 12219 | -0.650 | -0.1982 | Yes |
| 330 | SREK1 | splicing regulatory glutamic acid and lysine rich protein 1 [Source:HGNC Symbol;Acc:HGNC:17882] | 12221 | -0.651 | -0.1933 | Yes |
| 331 | U2AF2 | U2 small nuclear RNA auxiliary factor 2 [Source:HGNC Symbol;Acc:HGNC:23156] | 12226 | -0.652 | -0.1886 | Yes |
| 332 | CPSF7 | cleavage and polyadenylation specific factor 7 [Source:HGNC Symbol;Acc:HGNC:30098] | 12251 | -0.661 | -0.1855 | Yes |
| 333 | NCL | nucleolin [Source:HGNC Symbol;Acc:HGNC:7667] | 12255 | -0.662 | -0.1806 | Yes |
| 334 | CACTIN | "cactin, spliceosome C complex subunit [Source:HGNC Symbol;Acc:HGNC:29938]" | 12270 | -0.668 | -0.1766 | Yes |
| 335 | SUGP2 | SURP and G-patch domain containing 2 [Source:HGNC Symbol;Acc:HGNC:18641] | 12284 | -0.671 | -0.1725 | Yes |
| 336 | RBM28 | RNA binding motif protein 28 [Source:HGNC Symbol;Acc:HGNC:21863] | 12315 | -0.681 | -0.1697 | Yes |
| 337 | SRSF11 | serine and arginine rich splicing factor 11 [Source:HGNC Symbol;Acc:HGNC:10782] | 12322 | -0.683 | -0.1649 | Yes |
| 338 | AKAP17A | A-kinase anchoring protein 17A [Source:HGNC Symbol;Acc:HGNC:18783] | 12327 | -0.684 | -0.1600 | Yes |
| 339 | QKI | "QKI, KH domain containing RNA binding [Source:HGNC Symbol;Acc:HGNC:21100]" | 12340 | -0.688 | -0.1556 | Yes |
| 340 | HNRNPK | heterogeneous nuclear ribonucleoprotein K [Source:HGNC Symbol;Acc:HGNC:5044] | 12341 | -0.688 | -0.1504 | Yes |
| 341 | HNRNPA1 | heterogeneous nuclear ribonucleoprotein A1 [Source:HGNC Symbol;Acc:HGNC:5031] | 12356 | -0.693 | -0.1462 | Yes |
| 342 | SAFB2 | scaffold attachment factor B2 [Source:HGNC Symbol;Acc:HGNC:21605] | 12363 | -0.696 | -0.1413 | Yes |
| 343 | REST | RE1 silencing transcription factor [Source:HGNC Symbol;Acc:HGNC:9966] | 12382 | -0.703 | -0.1373 | Yes |
| 344 | CELF1 | CUGBP Elav-like family member 1 [Source:HGNC Symbol;Acc:HGNC:2549] | 12391 | -0.704 | -0.1326 | Yes |
| 345 | CLASRP | CLK4 associating serine/arginine rich protein [Source:HGNC Symbol;Acc:HGNC:17731] | 12394 | -0.705 | -0.1273 | Yes |
| 346 | SAFB | scaffold attachment factor B [Source:HGNC Symbol;Acc:HGNC:10520] | 12395 | -0.705 | -0.1219 | Yes |
| 347 | DHX16 | DEAH-box helicase 16 [Source:HGNC Symbol;Acc:HGNC:2739] | 12414 | -0.712 | -0.1179 | Yes |
| 348 | THRAP3 | thyroid hormone receptor associated protein 3 [Source:HGNC Symbol;Acc:HGNC:22964] | 12426 | -0.717 | -0.1132 | Yes |
| 349 | PABPN1 | poly(A) binding protein nuclear 1 [Source:HGNC Symbol;Acc:HGNC:8565] | 12461 | -0.730 | -0.1103 | Yes |
| 350 | TRA2A | transformer 2 alpha homolog [Source:HGNC Symbol;Acc:HGNC:16645] | 12479 | -0.740 | -0.1060 | Yes |
| 351 | THOC1 | THO complex 1 [Source:HGNC Symbol;Acc:HGNC:19070] | 12503 | -0.749 | -0.1021 | Yes |
| 352 | CCNT1 | cyclin T1 [Source:HGNC Symbol;Acc:HGNC:1599] | 12517 | -0.757 | -0.0973 | Yes |
| 353 | HNRNPH1 | heterogeneous nuclear ribonucleoprotein H1 [Source:HGNC Symbol;Acc:HGNC:5041] | 12554 | -0.776 | -0.0942 | Yes |
| 354 | PABPC1 | poly(A) binding protein cytoplasmic 1 [Source:HGNC Symbol;Acc:HGNC:8554] | 12562 | -0.778 | -0.0888 | Yes |
| 355 | PRPF40A | pre-mRNA processing factor 40 homolog A [Source:HGNC Symbol;Acc:HGNC:16463] | 12651 | -0.826 | -0.0895 | Yes |
| 356 | JMJD6 | "jumonji domain containing 6, arginine demethylase and lysine hydroxylase [Source:HGNC Symbol;Acc:HGNC:19355]" | 12658 | -0.832 | -0.0836 | Yes |
| 357 | AKAP8L | A-kinase anchoring protein 8 like [Source:HGNC Symbol;Acc:HGNC:29857] | 12665 | -0.836 | -0.0777 | Yes |
| 358 | SRRM2 | serine/arginine repetitive matrix 2 [Source:HGNC Symbol;Acc:HGNC:16639] | 12666 | -0.837 | -0.0712 | Yes |
| 359 | DAZAP1 | DAZ associated protein 1 [Source:HGNC Symbol;Acc:HGNC:2683] | 12686 | -0.854 | -0.0662 | Yes |
| 360 | THOC2 | THO complex 2 [Source:HGNC Symbol;Acc:HGNC:19073] | 12691 | -0.855 | -0.0599 | Yes |
| 361 | RBM6 | RNA binding motif protein 6 [Source:HGNC Symbol;Acc:HGNC:9903] | 12701 | -0.865 | -0.0540 | Yes |
| 362 | SFSWAP | splicing factor SWAP [Source:HGNC Symbol;Acc:HGNC:10790] | 12747 | -0.908 | -0.0506 | Yes |
| 363 | SRSF4 | serine and arginine rich splicing factor 4 [Source:HGNC Symbol;Acc:HGNC:10786] | 12749 | -0.911 | -0.0437 | Yes |
| 364 | RBBP6 | "RB binding protein 6, ubiquitin ligase [Source:HGNC Symbol;Acc:HGNC:9889]" | 12759 | -0.921 | -0.0374 | Yes |
| 365 | DDX39B | DExD-box helicase 39B [Source:HGNC Symbol;Acc:HGNC:13917] | 12783 | -0.941 | -0.0320 | Yes |
| 366 | SREK1IP1 | SREK1 interacting protein 1 [Source:HGNC Symbol;Acc:HGNC:26716] | 12810 | -0.988 | -0.0265 | Yes |
| 367 | SCAF4 | SR-related CTD associated factor 4 [Source:HGNC Symbol;Acc:HGNC:19304] | 12827 | -1.021 | -0.0199 | Yes |
| 368 | DDX5 | DEAD-box helicase 5 [Source:HGNC Symbol;Acc:HGNC:2746] | 12834 | -1.038 | -0.0124 | Yes |
| 369 | PRPF3 | pre-mRNA processing factor 3 [Source:HGNC Symbol;Acc:HGNC:17348] | 12837 | -1.040 | -0.0046 | Yes |
| 370 | ZFP36L1 | ZFP36 ring finger protein like 1 [Source:HGNC Symbol;Acc:HGNC:1107] | 12888 | -1.244 | 0.0010 | Yes |
Table: GSEA details [plain text format]

  

Fig 2: GOBP\_MRNA\_PROCESSING      
 Blue-Pink O' Gram in the Space of the Analyzed GeneSet

  

Fig 3: GOBP\_MRNA\_PROCESSING: Random ES distribution      
 Gene set null distribution of ES for **GOBP\_MRNA\_PROCESSING**
